# Iatrogenics in Orthodontics and its challenges

**DOI:** 10.1590/2177-6709.21.5.114-125.sar

**Published:** 2016

**Authors:** Gustavo Mattos Barreto, Henrique Oliveira Feitosa

**Affiliations:** 1 MSc in Orthodontics and Facial Orthopedics, Universidade de São Paulo, Araraquara, Brazil. Diplomate, Brazilian Board of Orthodontics and Facial Orthopedics.; 2 MSc in Orthodontics, Universidade Cidade de São Paulo (UNICID), São Paulo, São Paulo, Brazil.

**Keywords:** Iatrogenic disease, Orthodontics, Relapse

## Abstract

**Introduction:**

Orthodontics has gone through remarkable advances for those who practice it with dignity and clinical quality, such as the unprecedented number of patients treated of some type of iatrogenic problems (post-treatment root resorptions; occlusal plane changes; midline discrepancies, asymmetries, etc). Several questions may raise useful reflections about the constant increase of iatrogenics. What is causing it? Does it occur when dentists are properly trained? In legal terms, how can dentists accept these patients? How should they be orthodontically treated? What are the most common problems?

**Objective:**

This study analyzed and discussed relevant aspects to understand patients with iatrogenic problems and describe a simple and efficient approach to treat complex cases associated with orthodontic iatrogenics.

## RESUMO

**Introdução:**

a Ortodontia tem vivenciado momentos marcantes para quem a exerce com dignidade e qualidade clínica. Um deles é a chegada, sem precedentes, dos pacientes com algum tipo de iatrogenia, como: reabsorções radiculares pós-tratamento, alterações no plano oclusal, desvios de linha média, assimetrias, entre outras. Diversas questões permitem uma boa reflexão sobre esse constante aumento das iatrogenias. Qual o motivo? Acontecem com profissionais bem formados? Como receber, legalmente, esses pacientes? Como tratá-los ortodonticamente? Quais são os problemas mais comuns?

**Objetivo:**

o objetivo do presente trabalho é analisar e discutir pontos relevantes para melhor atender esse tipo de paciente e apresentar uma abordagem simples e eficaz na condução de casos complexos relacionados às iatrogenias ortodônticas.

## INTRODUCTION

### What are the causes of orthodontic iatrogenics?

Several reasons should be analyzed to explain the growing number of cases of iatrogenics in Orthodontics. Iatrogenics usually occurs due to inaccurate growth prediction, incorrect choice of orthodontic appliances, technical failure by the dentist, poor patient cooperation or lack of control of space and anchorage, particularly when teeth are extracted for orthodontic reasons.^1^

Iatrogenics may be described as a situation that leads to reversible or irreversible damage to patients that undergo any type of treatment. In 1996, Behrents^1^ defined iatrogenics as something unintentionally induced by treatment. All procedures involve a large number of relevant variables determined by patient characteristics, such as the dynamics of facial development and growth, the biomechanical interactions between appliances, dentition and bones, the dynamics of the dentist-patient-family interaction, the large variety of treatment approaches and the continuity of follow-up during the retention phase.^1^

Other difficulties in the conduction of a treatment may occur as a result of inadequate choice of dental procedures, incorrect treatment indication, adoption of hazardous treatment strategies, inadequate treatment performance, incorrect decision about treatment time, not changing treatment plan when necessary, not achieving a resolution of malocclusion, inappropriate follow-up during the retention phase, and not establishing good communication with the patient. All these failures may significantly affect outcomes, quality and stability of correction. Not performing the treatment adequately may result in a poor facial, gingival and dental outcome, and malocclusion correction and stability may be compromised and inadequate.^2^ Moreover, treatment time may be long, which may generate damage to teeth, pulp, support tissues, facial structures and general patient health^3^ ^,^ ^4^ ^,^ ^5^ This shows how complex and full of variables Orthodontics may be, which may lead to wrong strategies and, consequently, classical iatrogenics due to neglect in attending to all variables involved.

When iatrogenics is fully evaluated, we find good and bad aspects, and the latter are certainly predominant. The good aspects are associated with the margin of comparison that patients usually make between the previous and current treatments, assigning greater value to the second treatment when malocclusion is satisfactorily corrected. The bad aspects are associated with the lack of motivation to start a new treatment, which seriously affects cooperation. Patients are financially stressed and often demand very short treatment times, incompatible with the complexity and level of trauma of such cases.

There is no acceptable level of technical error, but we should be humble to recognize our possible professional errors and limitations. Dentists themselves should be able to try to solve possible problems honestly, avoiding greater complications that may arise from keeping a condescending attitude and not accepting the problem. We should never forget orthodontic relapses, which are and will always be present even in the offices of the most renowned orthodontists. Relapse should be evaluated carefully and not confounded with iatrogenics.^6^ Therefore, we should be guided by good sense in the first visit, so that re-treatment is not arbitrarily and hastily defined.

In such context, other questions about the same problem arise: Does iatrogenics occur when dentists are well trained? In legal terms, how can dentists accept treating these patients? How can they be treated orthodontically, and what problems are more common?

**Figure 1:**
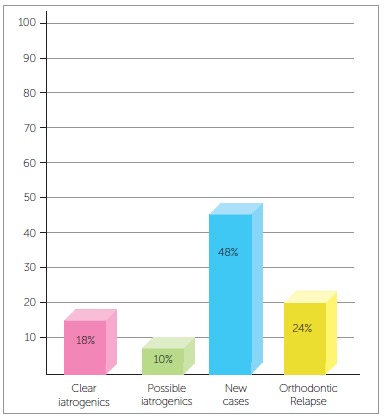
Percent distribution of 100 orthodontic examinations: clear iatrogenics (CI); possible iatrogenics (PI); new cases (NC); orthodontic relapse (OR).

To answer these questions, we have recently conducted a simple and objective survey in private clinics, which included one hundred orthodontic examinations made from April 10, 2014 to March 7, 2015. The examinations were analyzed by two orthodontists and divided into four groups: clear iatrogenics (CI) = clinical cases with problems clearly caused by another treatment; possible iatrogenics (PI) = clinical cases for which treatment plan was made by another dentist and that would probably result in iatrogenics; orthodontic relapse (OR) = clinical cases with loss of any amount of correction achieved in previous treatment; and new cases (NC) = cases that had not yet received any orthodontic treatment. Of the 100 cases evaluated, 18% were CI, 10% = PI, 24% = OR and 48% = NC, which demonstrated that a significant number of patients had iatrogenic problems (Fig 1). These findings indicated that over 50% of the patients in the study were directly affected by some problem: iatrogenics, possible iatrogenics or relapse. Therefore, all the questions listed above have to be addressed clearly and completely, without masking facts, and the discussion has to be conducted by several groups, such as clinical orthodontists and academic staff, to produce improved results and rebuild credibility in orthodontic treatments.

### In legal terms, how can dentists accept treating these patients? 

The first visit should include clear explanations to the patient, and the dentist should be realistic, yet optimistic. We have to face the future optimistically and, at the same time, avoid turning visits into opportunities for the expression of endless grievances and excessive criticism of previous treatments. The first contact with patients, during the first visit, is at the receptionist's desk, where as much information as possible should be gathered about the previous use, or not, of appliances. When these patients come into our offices, we should conduct the visit according to the information collected. However, we should avoid, above all else, knowing who their previous dentist was. This will ensure that the visit is impartial and honest, and the dentist will know how to carefully evaluate whether re-treatment should address relapse or the sequelae of previous treatments. These patients are usually emotionally and financially stressed, poorly motivated and skeptical of Orthodontics. We often find out the name of the other dentist, but should not abstain from conducting an impartial and honest evaluation, as ethics is a direct driver of health promotion.

**Figure 2:**
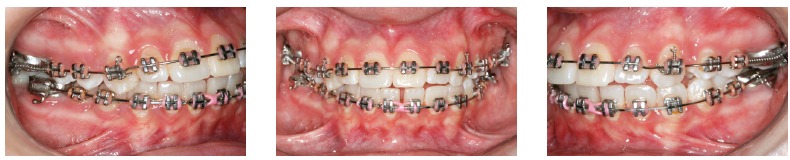
Intraoral photographs showing the orthodontic appliance used in previous treatment.

**Figure 3:**
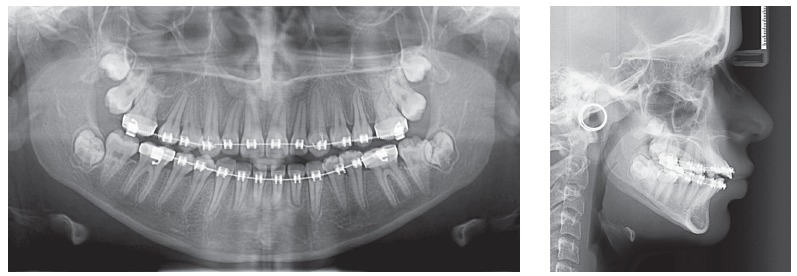
Panoramic and lateral radiographs showing the orthodontic appliance used in previous treatment.

**Figure 4:**
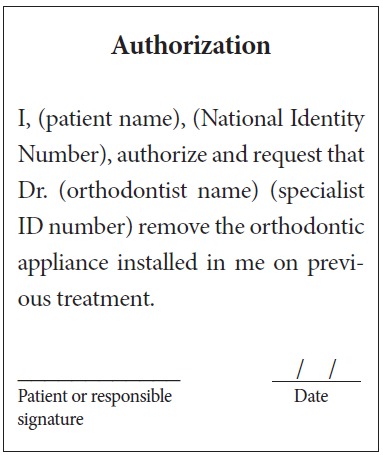
Authorization to remove orthodontic appliance.

**Figure 5:**
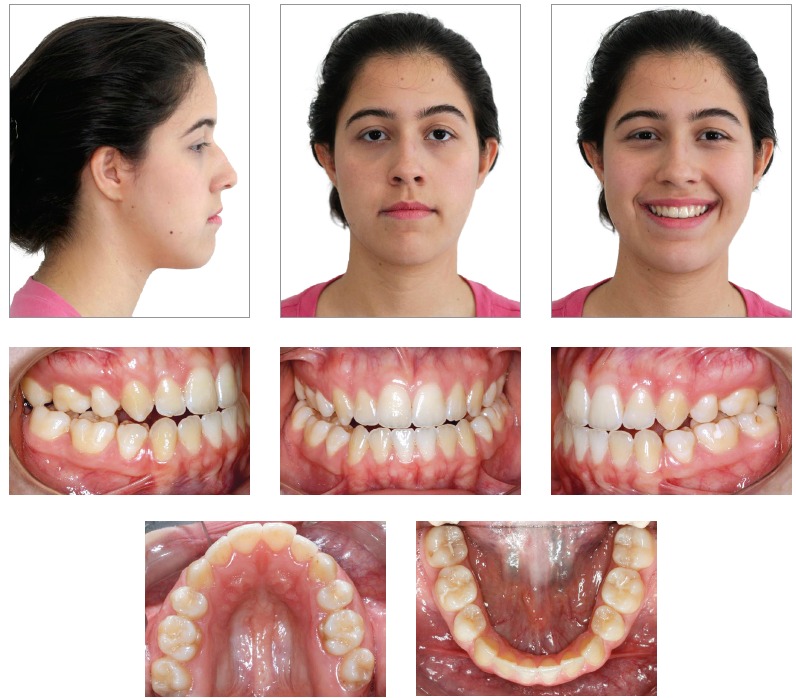
Photographs of clinical conditions at the beginning of treatment.

Orthodontic records of patients with iatrogenic problems should be impeccable. Complete photographic and radiographic records should be obtained when the orthodontic appliance is still in the mouth (Figs 2 and 3). After that, the problem of who should remove the appliance should be tackled. In the past, we used to try to convince the patient that the best treatment option would be to have the appliance removed by the dentist that had originally placed it. We found, however, a complete lack of commitment by other dentists, who did not remove the resin completely, which resulted in actual damage to enamel. Another setback was patient reluctance to return to the previous dentist because of emotional distress. Therefore, the ideal solution is to change the treatment protocol and remove the appliance, as long as the patient signs an authorization term (Fig 4). After removal, study models and new photos should be obtained without any appliance in the mouth, to prepare for the new treatment (Fig 5). In this new treatment, the orthodontist should make all treatment options very clear, so that the patient is not deceived by any ideas of a "miraculous" treatment and accept the new orthodontic approach.

### Does iatrogenics occur when dentists are well trained? 

Every time iatrogenics is discussed, there is a partly justifiable concern that short Dentistry courses offering inadequate training are associated with cases of patients that come to our offices with iatrogenic problems, particularly with complaints about treatment timing, lack of effective results and no improvement during the treatment. Although such ideas and correlations make sense, we should not forget that we have also seen patients with some degree of iatrogenics who were originally treated by dentists trained according to all required standards - a fundamental fact when discussing this problem. According to Greco,^7^ the large number of orthodontics training courses has resulted in dentists whose attitudes, not consistent with health promotion, seriously affect patients.

**Figure 6:**
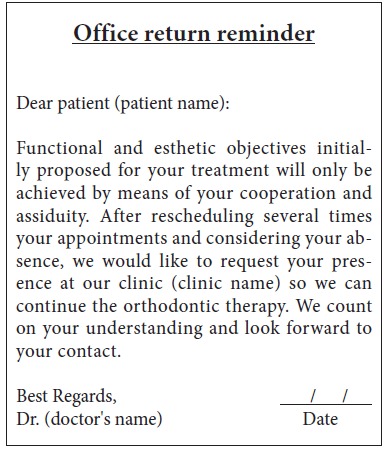
Office return reminder.

The organization of our care services should be classified at the same level of importance of an adequate training. We are clearly living in an era of extreme technological advances and very useful resources, but we should know how to take advantage of these essential aspects, not forgetting the basis of a solid and successful treatment. Some organization requisites deserve special attention: 

1) The number of patients that we have: according to Kokich^8^, we should keep track of how many patients start and how many complete their treatments in our offices. This is the only way to keep our quality standards and assign the necessary time to treat each patient. If we do not keep clear records, we will have offices crowded with people and will lose control of patient numbers, which may lead to poor quality of treatment results. 

2) Time for diagnosis and planning. Our involvement with clinical time often leads us to neglecting fundamental steps of diagnosis and treatment plans, which are undoubtedly the pillars of successful treatments. 

3) Absences control. It is fundamental to control whether our patients do not show up or return, because this may, in fact, be responsible for consequences to the most renowned orthodontists.^9^ Imagine a patient that has not returned, but who has continued using intermaxillary elastics without any interruption. To avoid that, a reminder, particularly the type that requires a return receipt by physical or digital mail, should be sent to all patients (Fig 6). 

4) Other points that should be kept in mind and adopted are: appointments should be made so that the orthodontist has time to do what was planned for that visit; the dentist should have time to treat complications as soon as possible, as well as time to re-study cases using radiographs and photos, so that problems are controlled; and, finally, good records should be kept after the end of the treatment. These measures are fundamental to avoid legal problems and, in extreme cases, to defend against possible accusations. We should remember that Orthodontics has been implicated in recurrent legal suits. 

### What are the most common problems and how to treat them?

According to data in Figure 1, which shows the numbers of iatrogenic problems, these cases are closely associated with treatments that include extractions for therapeutic reasons: 75% of the patients with iatrogenic problems presented tooth extractions in their treatment plan, whereas 13% were treated without extractions and 12% had other problems. These data demonstrate that orthodontists, in general, have difficulties in treating cases that require extractions and anchorage control, cases that often involve excessive forces in inadequate directions.^10^ An example of this is the clinical case of a 28-yer-old patient who presented with a complaint of severe midline discrepancy after orthodontic treatment that lasted 9 years. Two maxillary premolars had been extracted for orthodontic correction. The panoramic radiograph showed an unexpectedly advanced degree of tooth resorption (Fig 9). As the patient had the baseline panoramic radiograph for the first treatment, we could compare the root structures before and after that treatment (Fig 10).

**Figure 7:**
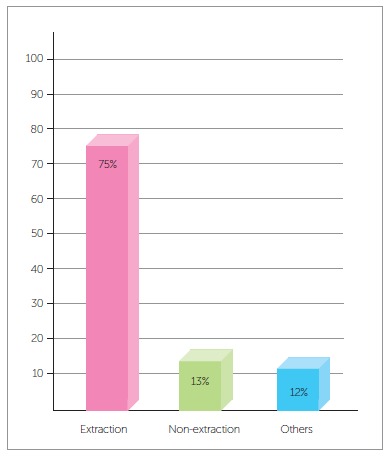
Percent distribution of orthodontic iatrogenics.

**Figure 8:**
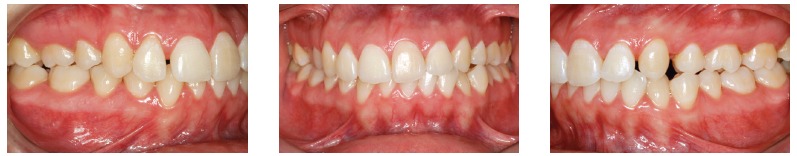
Photographs of clinical conditions at the beginning of treatment.

**Figure 9:**
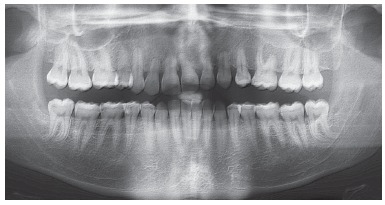
Baseline panoramic radiograph.

**Figure 10:**
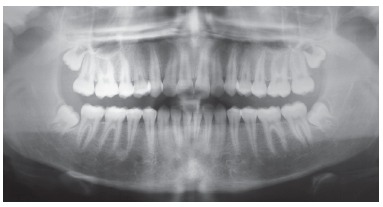
Baseline panoramic radiograph obtained before first orthodontic treatment.

**Figure 11:**
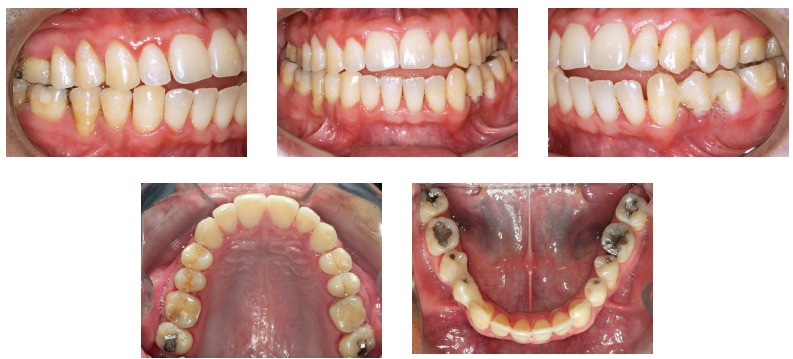
Photographs of clinical conditions at the beginning of treatment.

**Figure 12:**
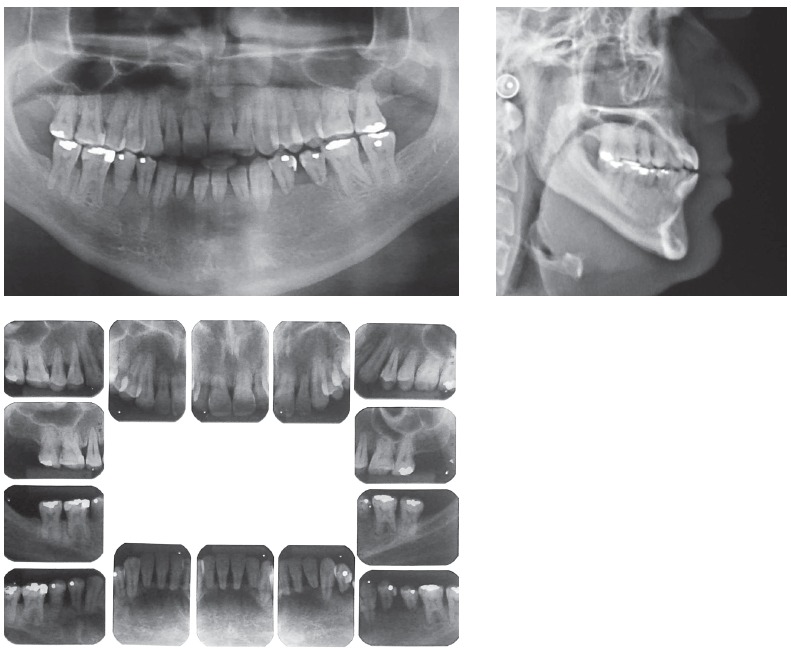
Baseline panoramic, periapical and lateral radiographs.

**Figure 13:**
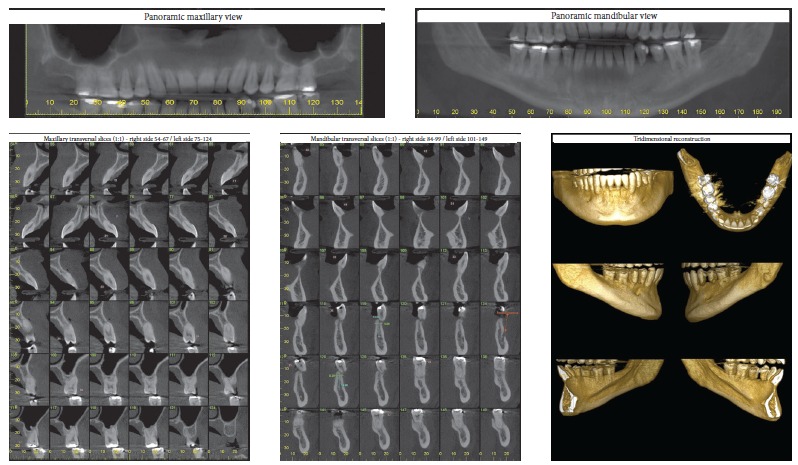
Baseline cone-beam CT scan.

**Figure 14:**
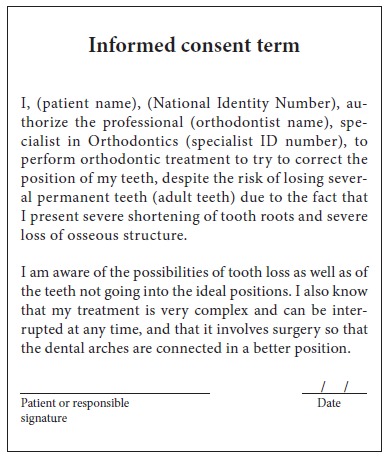
Personalized informed consent term to start treatment.

According to Marques,^11^ in cases of root resorption, root shapes should be carefully evaluated, particularly when premolars have to be extracted, because this extraction increases the risk of severe root shortening. Moreover, extensive root resorption induced by orthodontic treatment may be associated with predictability, prevention and early diagnosis, and are clear signs of the success or the failure of orthodontic treatments.^12^ We should be prepared to treat patients with iatrogenic problems, and, because of that, an informed consent term should be part of our routine. However, it should be undoubtedly personalized for each case, so that the treatment is clearly defined, which will make both patient and dentist feel at ease and safe. This situation is show on the case presented in Figures 11 to 13, of a 39-year old patient that was unhappy with the result and duration (5 years) of his treatment illustrates this point. The clinical evaluation revealed an anterior open bite, canted occlusal plane, posterior crossbite and buccal inclination of mandibular incisors (Fig 11). Baseline tests revealed a significant degree of tooth mobility, which might be assigned to root resorption. Lateral, panoramic and periapical radiographs of maxillary and mandible surprisingly revealed much greater root and bone resorption than expected, involving practically all teeth, but more severe in mandibular teeth (Fig 12). For a more detailed analysis, a CT scan was obtained, and findings confirmed tooth and bone resorption (Fig 13). After that, the complexity of all procedures was carefully explained to the patient, because some of the teeth would definitely have to be extracted and some others might also have to be extracted during the treatment. At this point in the treatment, the patient received a personalized informed consent term that clearly stated all possible procedures and risks involved in the treatment (Fig 14). In cases when the patient refuses to sign the informed consent term, the dentist should refuse to conduct the treatment, because we should prevent and be protected against possible complications.

**Figure 15:**
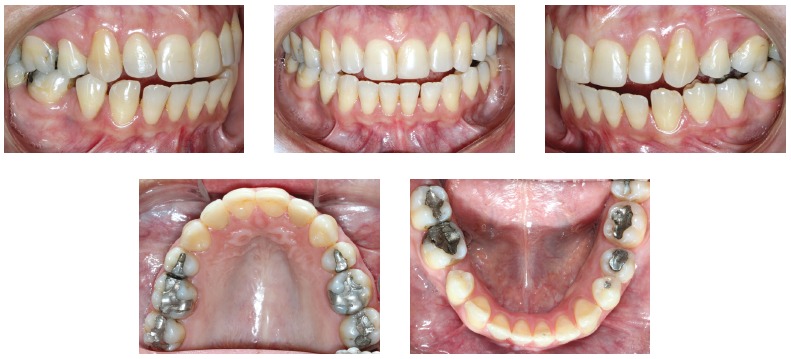
Photographs of clinical conditions at the beginning of treatment.

**Figure 16:**
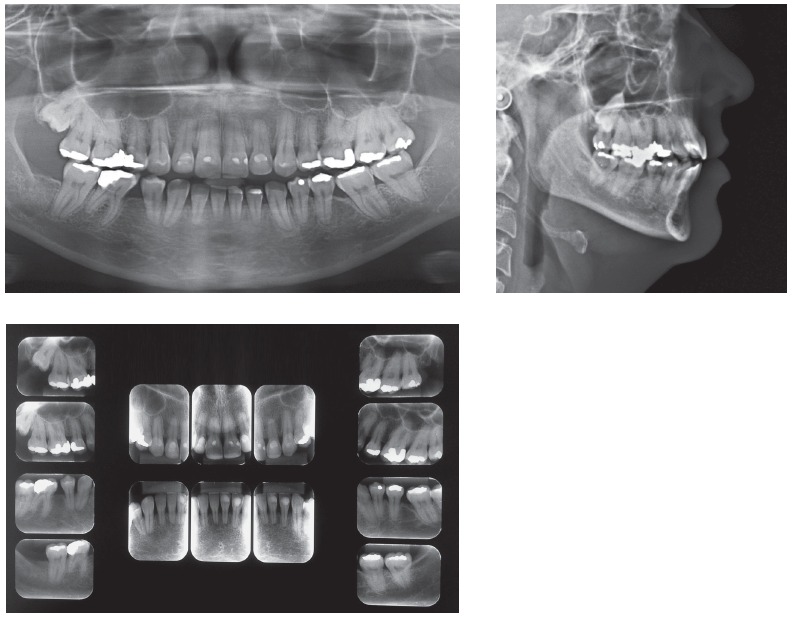
Baseline panoramic, periapical and lateral radiographs.

The clinical case presented in Figures 15 and 16 illustrates a case that involved tooth extractions. A 39-year-old patient sought orthodontic treatment to correct malocclusion that persisted four years after a previous treatment. Baseline examination revealed moderate anterior open bite and the absence of three first premolars, which, according to the patient, had been extracted during the first treatment. The spaces opened by extractions and the strong articular pain that the patient felt were greatly distressing (Figs 15). For an accurate diagnosis, periapical, panoramic and lateral radiographs were obtained. Their analysis revealed significant root resorption associated with severe mobility of mandibular teeth (Fig 16). Following the recommended protocol, treatment limitations were explained to the patient, who was asked to sign a personalized informed consent term, similar to that shown in Figure 14. After that, the treatment plan was followed, and both patient and dentist were aware of all possible outcomes. 

**Figure 17:**
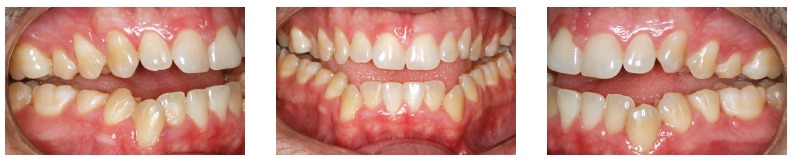
Baseline intraoral photos with no mandibular manipulation.

**Figure 18:**
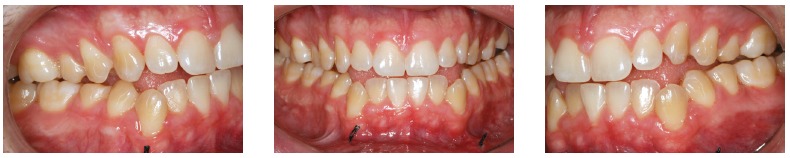
Baseline intraoral photographs with mandibular manipulation.

**Figure 19:**
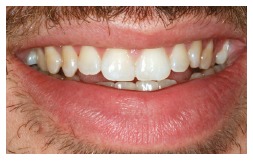
Baseline close-up smiling photograph without mandibular manipulation.

**Figure 20:**
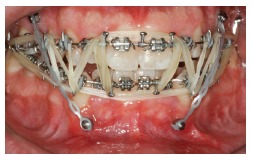
Intraoral photograph with intermaxillary fixation, by means of a passive orthodontic appliance.

**Figure 21:**
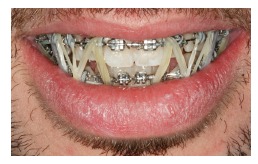
Close-up smiling photograph with intermaxillary fixation.

**Figure 22:**
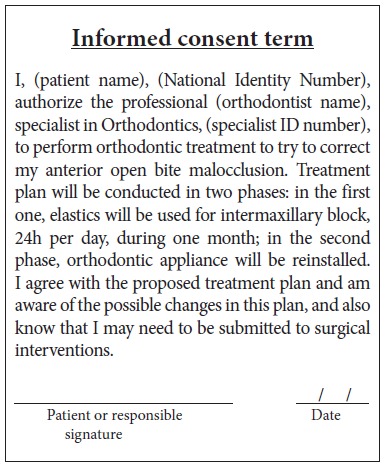
Personalized informed consent term

In another example of the use of a personalized informed consent term, a 31-year-old patient (Figs 17 to 21) sought urgent care in an orthodontic office to treat a problem resulting from an orthognathic surgery one month before, as part of the first treatment. During the examination, the patient was greatly concerned because his mandible was loose, a characteristic of post-surgical relapse after intermaxillary fixation removal. It is noteworthy thathe patient was not using an orthodontic appliance as part of that surgical treatment. Clinically, the patient presented with two bite patterns, as the mandibular osteotomy was not consolidated: in the first pattern, without manipulation, the mandible was advanced, creating a Class III relation, with severe anterior open bite (Fig 17); in the second one, a better relation between arches was achieved when the mandible was positioned backwards (Figs 18 and 19). We immediately decided to mount the appliance passively to reduce fracture and avoid a second surgery (Figs 20 and 21). At that time, we explained the treatment, answered questions and asked the patient to sign a personalized informed consent term, as shown in Figure 22. 

The clinical cases described herein highlight the importance of adequate and detailed planning of high-complexity orthodontic procedures involving iatrogenics and, above all, the importance of knowing how to deal with these real and frequent situations in dental offices. We should know how to use our technical and emotional skills to adopt an ethic approach, but should not miss the fact that the main characteristic of our practice is to provide quality healthcare to our patients.

## CONCLUSIONS

We should be prepared to treat patients with iatrogenic problems, and one of our principal instruments should be the personalized informed consent term, adopted to ensure that treatments are clear and transparent. We should also always have in mind our limitations, the importance of good orthodontic training and, particularly, the extra value that should be assigned to the organization of our healthcare services. This is probably the only way we may decrease the chances of being responsible for orthodontic iatrogenics.
